# Ventilation approaches and radon control in Canadian houses

**DOI:** 10.3389/fpubh.2025.1569494

**Published:** 2025-04-10

**Authors:** Janet Gaskin, Liang Grace Zhou, Yunyi Ethan Li, Patrique Tardif

**Affiliations:** Construction Research Centre, National Research Council Canada, Ottawa, ON, Canada

**Keywords:** radon, exposure, mitigation, ventilation, heat recovery ventilation, depressurization, dwellings

## Abstract

**Introduction:**

Balanced mechanical ventilation with heat (sensible heat only) or energy (sensible and latent heat) recovery has the potential to dilute indoor radon and may be an appropriate first step at reducing moderate indoor radon concentration in a house with mechanical exhaust only. A field study of the effectiveness of heat/energy recovery ventilation systems at reducing moderate indoor radon concentration was conducted in 13 occupied houses and three test houses in Canada. Controlled experiments were also conducted at the test houses to evaluate indoor radon concentration under different depressurization and ventilation scenarios.

**Methods:**

In the field study of heat recovery ventilation systems (HRVs) in occupied homes, the indoor radon concentration was measured for different HRV settings within a season to estimate the effectiveness of radon reduction. In the controlled depressurization experiments in the test houses, the change in pressure of the basement relative to the subslab and of the basement, main floor and second floor relative to outdoors was evaluated for a range of mechanical exhaust ventilation scenarios.

**Results:**

The initial radon concentrations measured on the lower floor in the study houses with the HRV off were moderate, ranging from 91 to 312 Bq/m^3^, with a median of 175 Bq/m^3^. The median (25th–75th percentiles) effectiveness of radon reduction was 39% (29–50) for continuous HRV operation at high fan speed in the 12 field study houses where it was connected directly to the forced air furnace heating systems. In the test houses, the sustained operation of mechanical exhaust ventilation devices, however, increased the depressurization and the indoor radon concentration, and the indoor radon concentrations observed at the depressurization of −5 Pa were higher than those observed at −10 and −20 Pa.

**Conclusion:**

Balanced mechanical ventilation with heat recovery was shown to be an effective radon control strategy in a field study of occupied houses with a moderate initial indoor radon concentration. Improving the energy efficiency of the existing housing stock is a priority in many countries, and including balanced mechanical ventilation systems may be an effective radon control option when conducting energy retrofits in existing houses.

## 1 Introduction

Radon is a naturally occurring radioactive soil gas that is formed as uranium decays, and the infiltration of soil radon gas through building foundations can result in increased radon concentrations indoors. Radon ingress can occur by convection through any openings in the foundations as a result of pressure differentials and by diffusion across an airtight barrier. The World Health Organization recommends that national radon programs reduce both the population exposure to radon and the exposure of individuals at highest risk (1). About 3,000 lung cancer deaths a year are attributed to long-term exposure to residential radon in Canada, resulting in radon being considered the second leading cause of lung cancer for Canadians after smoking (2, 3). The Health Canada guideline recommends mitigating average annual indoor radon concentrations above 200 Bq/m^3^ in existing housing and suggests residents may choose to mitigate lower radon exposures because any reduction in indoor radon will decrease the associated lung cancer risk (4). Health Canada also suggests installing balanced mechanical ventilation may be a mitigation option for existing houses when only a modest reduction in indoor radon is required. Low-rise dwellings are the most common structural type in the Canadian housing stock reported in the 2021 census, with single detached houses representing 53% of all private dwellings, and low-rise housing that includes single detached, duplex and townhouses, representing 70% of all private dwellings (5).

Residential ventilation systems and exhaust devices can increase indoor radon concentrations if they result in depressurization of the conditioned spaced relative to the ground or to outdoors. Balanced mechanical supply and exhaust ventilation systems tend to dilute the indoor radon concentration, although some depressurization of the lower level can result from the air distribution if air is primarily supplied to upper level rooms and exhausted from lower level rooms. A heat (sensible heat only) or energy (sensible and latent heat) recovery ventilation system intends to provide balanced mechanical supply and exhaust airflows, and includes a heat or energy exchange core to reduce the energy loss associated with replacing stale indoor air with outdoor air that must be either cooled or heated to maintain thermal comfort. Mechanical exhaust only ventilation systems depressurize the conditioned space and rely on the resulting infiltration of air across the building envelope that can occur both above and below grade. Higher indoor radon concentrations have been reported for the larger depressurization of 5–9 Pa in houses with mechanical exhaust only ventilation systems relative to 2–3 Pa in houses with balanced mechanical supply and exhaust ventilation systems (6).

Two municipalities in Sweden reported decreased indoor radon in older housing in which the ventilation system had been improved (7). Lower indoor radon concentration was reported for low-rise housing that included balanced mechanical ventilation systems compared to those with exhaust only ventilation in Finland, Norway and Sweden (6, 8–11). In Japan, significantly reduced indoor radon was reported for houses built after 2003, when the building code was changed to require a balanced mechanical ventilation system be installed in new houses to keep formaldehyde concentrations below the guidelines value and prevent “sick building” syndrome (12). A survey of 200 newly constructed houses in Denmark reported indoor radon measurements, and evaluated the association between indoor residential radon levels and housing characteristics (13). Tracer gas testing to evaluate the air exchange rates was restricted to the 20 houses that had the highest indoor radon concentrations, representing 10% of the houses recruited, due to time and economic restraints. Although the median ACH reported for these 20 homes was 0.3/h, higher indoor radon was not found to be significantly associated with lower air exchange and the two houses with the highest radon were not those with the lowest air exchange rates.

A field study of the effectiveness of heat recovery ventilation systems (HRV) at reducing moderate indoor radon concentration (below about 300 Bq/m^3^), by evaluating different HRV settings in each house, was conducted for 16 existing houses between the winter of 2020–2021 and 2022–2023 in the National Capital Region in Canada. Controlled experiments were also conducted at the Canadian Centre for Housing Technology (CCHT) to evaluate indoor radon concentration under different depressurization and ventilation scenarios. The depressurization resulting from typical exhaust devices was evaluated, and indoor radon concentrations were measured during depressurization testing using a duct blaster.

## 2 Materials and methods

### 2.1 Field study of radon in occupied housing following HRV retrofits

The recruitment of field study houses was restricted to those in which moderate initial radon concentrations were measured. The characteristics of the study houses are described in Table 1, including house type, number of floors (excluding the basement), the presence/absence of a basement, the type of heating system, the type of balanced mechanical ventilation system and whether the house has a passive soil depressurization (PSD) system installed for radon mitigation. HRV systems were retrofitted in 13 occupied houses in the community that lacked balanced supply and exhaust mechanical ventilation systems, houses A to M in Table 1. The mechanical ventilation systems were balanced following installation, with the airflows at the high fan speed ranging from 65 to 80 cfm, supplementing the existing exhaust capacity of the bathroom fans vented directly outdoors. The total ventilation capacity in these houses therefore exceeded the minimum recommended in the Canadian standard for residential ventilation, CSA F326 (14), which is based on estimated occupancy and calculated using the number and type of rooms in the dwelling. The HRV supply and exhaust were both connected to the forced-air furnace return duct in the 12 study houses that had forced-air furnaces for heating, while an independently ducted HRV was installed for the house with electric baseboard heating. The field study testing also included three National Research Council (NRC) experimental houses in which energy recovery ventilation systems were already installed. The NRC Canadian Centre for Housing Technology (CCHT) full size, side-by-side, energy efficient twin duplexes, CCHT West and CCHT East, were designed to the R-2000 standard (15) with simulated occupancy, which means that appliances, lighting, plumbing and equipment all run as though the houses were occupied by a family of four. The energy recovery systems installed in CCHT West and East have independently ducted exhaust from the kitchen and bathrooms and the supply connected to the return of the forced-air furnace. The third NRC house was a smaller experimental test house, in which independently ducted HRV was installed. All of the study houses have a basement, and most have a forced air furnace for heating, while two had electric heating. The study houses included one and two-story detached houses, and two-story semi-detached and townhouses. Half of the houses had a PSD system installed for radon mitigation.

**Table 1:** Study house characteristics.

House ID	House type	# Floors	Basement	Heating type	Balanced mechanical ventilator	Passive soil depressurization
A	Town	2	✓	Forced-air furnace	HRV	x
B	Town	2	✓	Forced-air furnace	HRV	x
C	Det	2	✓	Forced-air furnace	HRV	✓
D	Det	1	✓	Forced-air furnace	HRV	✓
E	Det	1	✓	Forced-air furnace	HRV	x
F	Det	2	✓	Forced-air furnace	HRV	✓
G	Det	2	✓	Forced-air furnace	HRV	x
H	Det	2	✓	Forced-air furnace	HRV	✓
I	Det	2	✓	Forced-air furnace	HRV	✓
J	Det	2	✓	Forced-air furnace	HRV	x
K	Det	2	✓	Forced-air furnace	HRV	x
L	Det	2	✓	Forced-air furnace	HRV	x
M	Det	1	✓	Electric baseboard	HRV	x
CCHT West	Semi-det	2	✓	Forced-air furnace	ERV	✓
CCHT East	Semi-det	2	✓	Forced-air furnace	ERV	✓
Experimental house	Det	1	✓	Electric heater	HRV	✓

The indoor radon concentration was measured every hour on both the lower and upper floors using continuous radon monitors (Corentium Pro) in all the study houses. The Corentium Pro monitors were calibrated annually, as recommended, using an independent third-party service. The average indoor radon concentration was calculated over roughly a one-month period for each HRV setting evaluated in the occupied houses, with testing conducted sequentially within a single season for each study house. The study spanned a two-year period, from the winter of 2020–2021 to 2022–2023, with the measurement dates for each study house listed in Table 2. The HRV settings evaluated included the following: off, operating continuously or periodically (typically 20 min per hour), and with a higher or lower fan speed when running. The airflows with the fan running at the lower speed ranged from around 40–50 cfm. Shorter two-week periods of radon measurement were conducted in the highly controlled energy efficient NRC twin duplex houses for each ventilation system setting. In four occupied houses in the community, the indoor radon concentrations for different HRV settings were measured both independently and in combination with the PSD system installed, as shown in Table 2. The residents were asked to maintain closed house conditions during the testing.

**Table 2:** Radon concentrations measured on lower and upper floors, with HRV off and operated continuously (average radon concentration over the measurement period ± measurement error).

House ID	Season	Time period	PSD installed and operating	Radon L floor HRV off (Bq/m^3^)	Radon L floor HRV cont. (Bq/m^3^)	Radon U floor HRV off (Bq/m^3^)	Radon U floor HRV cont. (Bq/m^3^)
A	Winter	2020-12-23/2021-03-03	x	125 ± 8	77 ± 6	125 ± 8	70 ± 6
B	Winter	2020-12-23/2021-03-13	x	91 ± 7	73 ± 6	75 ± 6	57 ± 5
C	Winter	2021-01-16/2021-03-15	x	127 ± 8	97 ± 7	123 ± 8	99 ± 7
C	Spring	2021-04-19/2021-06-15	✓	26 ± 3	17 ± 3	–	–
D	Spring	2021-01-29/2021-04-07	x	208 ± 12	101 ± 7	156 ± 10	77 ± 6
D	Fall	2021-11-06/2022-01-01	✓	110 ± 8	61 ± 5	78 ± 6	49 ± 4
E	Spring	2021-01-28/2021-04-02	x	104 ± 7	57 ± 5	98 ± 7	52 ± 5
F	Spring	2021-02-17/2021-04-19	x	309 ± 17	162 ± 10	174 ± 11	140 ± 9
F	Summer	2021-05-19/2021-07-26	✓	119 ± 8	43 ± 4	80 ± 6	34 ± 4
G	Spring	2021-03-19/2021-05-26	x	109 ± 7	58 ± 5	54 ± 5	24 ± 3
H	Summer	2021-05-17/2021-07-16	✓	134 ± 9	57 ± 5	108 ± 7	50 ± 5
I	Fall	2021-10-29/2021-12-24	✓	217 ± 13	164 ± 10	174 ± 11	150 ± 10
J	Winter	2022-11-04/2023-01-06	x	220 ± 13	108 ± 7	217 ± 13	91 ± 7
K	Winter	2022-11-21/2023-01-16	x	241 ± 14	177 ± 11	183 ± 11	94 ± 7
L	Winter	2022-11-29/2023-02-11	x	104 ± 7	61 ± 5	50 ± 5	37 ± 4
M	Spring	2021-03-26/2021-05-27	x	259 ± 15	68 ± 5	150 ± 10	14 ± 3
CCHT West	Winter	2021-01-08/2021-02-19	x	184 ± 11	65 ± 5	–	–
CCHT East	Winter	2021-01-08/2021-02-19	x	166 ± 10	95 ± 7	181 ± 11	92 ± 7
Experimental house	Winter	2021-02-10/2021-04-07	x	312 ± 18	171 ± 7	299 ± 17	160 ± 7

### 2.2 Mechanical exhaust ventilation and depressurization testing at the Canadian Centre for Housing Technology

**Figure 1:**
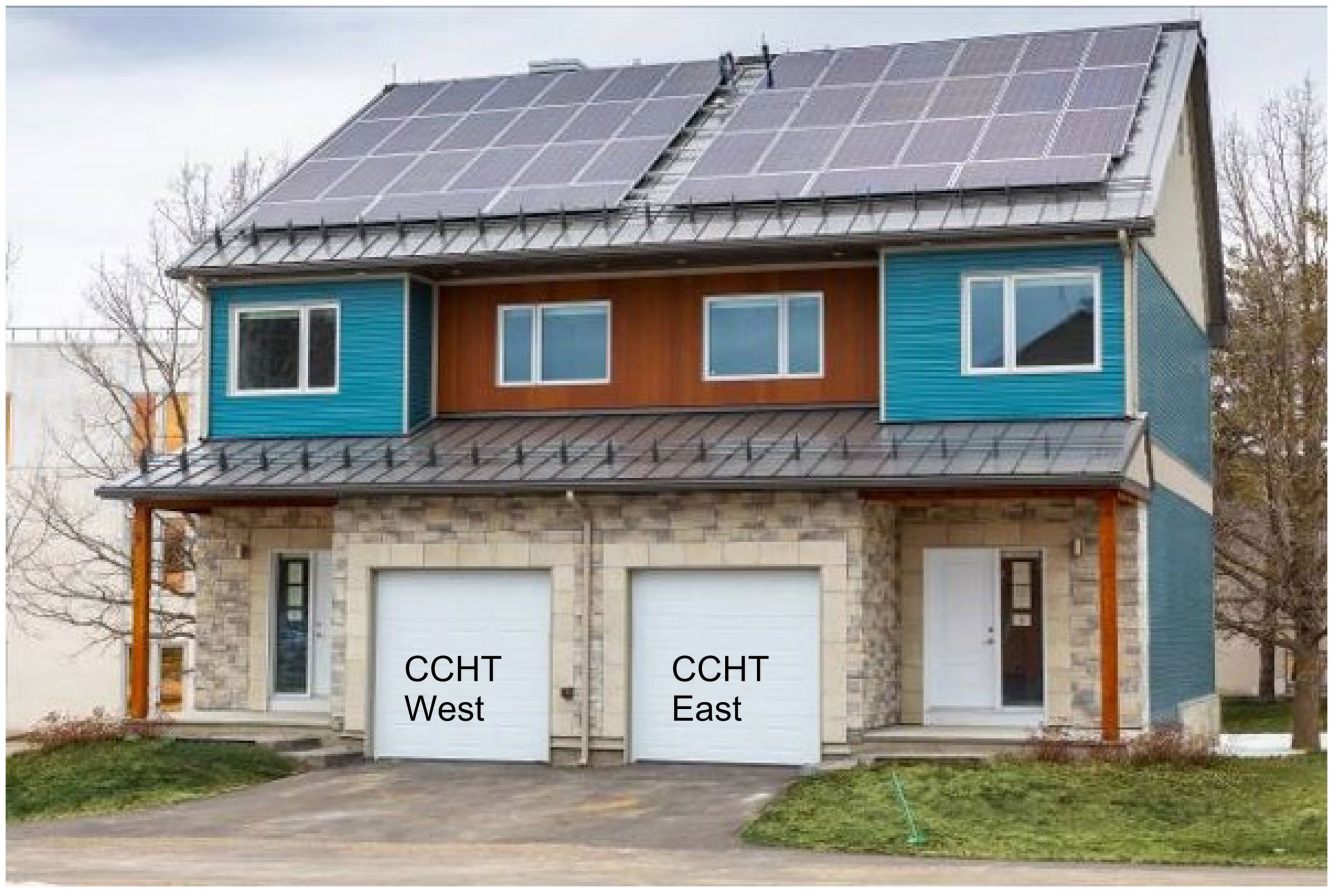
Canadian Centre for Housing Technology (CCHT) semi-detached twin houses, CCHT West and CCHT East (NRCC).

The NRC has multiple research houses located in Ottawa, Canada, and enables testing and monitoring of building technologies in full sized Canadian housing. The depressurization testing was conducted in the CCHT full-size two story net-zero energy-ready twin duplex houses (mirrored about the party wall) shown in Figure 1, which were constructed to the Canadian 2012 R-2000 standard that requires guarded testing of air change per hour at 50 Pa no greater than 1.5 air changes per hour (15). Both houses had a natural gas furnace, a central air conditioner, and an energy recovery ventilator. A duct system distributed conditioned outdoor air and re-circulated air throughout the houses. A 20-mil polyethylene radon barrier was installed during the construction of the basement and foundation. Guarded airtightness testing was conducted in 2021 and reported 1.46 air changes per hour at 50 Pa for CCHT West and 1.37 for CCHT East (16). Depressurization testing was also conducted in an older, smaller NRC experimental house with a main floor and basement that had a 6-mil polyethylene membrane installed beneath concrete slab foundation as a vapor barrier. Airtightness testing conducted with the HRV intake and exhaust sealed reported 3.6 air change per hour at 50 Pa. This test house had plug-in electric heaters, a split air conditioning unit, an independently ducted HRV, and no exhaust devices. Pressure differentials were measured using Setra model 264 low pressure differential transducers (±0.25 inch W.C., error ± 1% FS). Indoor radon concentrations were measured using continuous radon monitors (CRMs) every 10 min (AlphaGuard DF2000 Pro), on each floor in the semi-detached houses as shown in Supplementary Figure 1, and using 5 CRMs in the experimental test house as shown in Supplementary Figure 2. The AlphaGuard monitors were calibrated every 5 years, as recommended, using an independent third-party service.

The change in pressure from the operation of mechanical exhaust devices in CCHT West relative to the baseline condition when no devices were operating was evaluated in both CCHT energy efficient twin duplex houses from October 13–14, 2021. The mechanical exhaust devices included the bathroom exhaust fans, a powder room on the main floor and a main bathroom and an ensuite bathroom on the second floor, the range hood in kitchen, and the clothes dryer. The change in pressure of the basement relative to the subslab and of the basement, main floor and second floor relative to outdoors was evaluated during a 30-min period for each mechanical exhaust ventilation scenario, for exhaust devices operated individually, and with multiple exhaust devices operated simultaneously. The clothes dryer was also evaluated for two different locations, in the basement and on the second floor, as shown in Supplementary Figure 3.

To evaluate the indoor radon concentration at increased levels of depressurization, a duct blaster was installed in the door between the house and the garage on the main floor in CCHT East to exhaust to the outdoors. The impact of increasing the depressurization of the main floor relative to the outdoors using the duct blaster was evaluated between September 17 and October 13, 2021. Prior to the depressurization testing, the ventilation system and exhaust devices were turned off and sealed in both test houses. The furnace and the recirculation fan were turned off in the semi-detached house in the first round of testing and running using the larger ductwork in the semi-detached houses during the second round of testing. The indoor radon concentration and change in pressure relative to the baseline conditions were measured during a 48-h period on each floor of the duplex houses for increases in depressurization of −5 Pa, −10 Pa, and −20 Pa relative to the outdoors. The change in pressure between the basement and the subslab area was also measured. Similar depressurization tests were carried out in the older and smaller experimental house. This house had plug-in electric heating only and no recirculation, and the HRV was turned off and sealed during the depressurization tests.

## 3 Results

### 3.1 Radon reductions in occupied housing with HRV retrofits

The initial indoor radon concentrations measured on the lower and upper floors for the 16 study houses were presented in Table 2, both with the HRV off and while operating continuously. The initial radon concentrations measured on the lower floor in the 16 study houses with the HRV off were moderate, ranging from 91 to 312 Bq/m^3^, characterized by a median (25th – 75th percentile) of 175 (117–219) Bq/m^3^. Two testing scenarios were evaluated for several study houses in which both an HRV and a PSD system were installed, for the HRV only and for the combination of the HRV and the PSD. The indoor radon concentrations reported in Table 2 were measured for continuous HRV operation at high fan speed to represent the optimal radon reduction under normal occupancy conditions.

**Figure 2:**
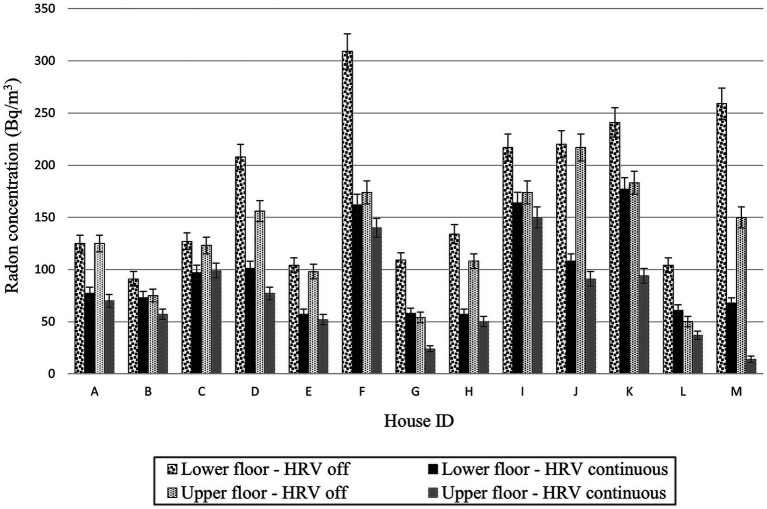
Radon concentrations measured in occupied study houses, by floor and HRV status.

The radon concentrations for the HRV off and the HRV operated continuously at the high fan speed for the 13 occupied houses in the community were shown in Figure 2. The radon concentrations on both the lower and upper floors were reduced in each house by the operation of the HRV, although the effectiveness of the radon reduction varied between houses. The radon concentration was somewhat higher on the lower floor than the upper floor for each HRV scenario for house A to house L, where the operation of the forced-air furnaces was mixing the air. Figure 2 showed that much higher radon was measured in the basement than on the first floor of the house with electric baseboard heating, house M.

The ratio of the radon concentration measured on the upper floor relative to the lower floor was plotted for each occupied field study house with the HRV off and operating continuously in Supplementary Figure 4. In houses with forced-air furnace heating, houses A to L, there was little change in radon stratification between the upper and lower floors when the HRV was operated continuously; in some of these houses the air was very well mixed and the ratio of radon concentration on the upper to lower floor was close to 1, while in others a lower ratio indicated greater radon stratification. The ratio of radon concentration on the upper floor relative to the lower floor was much lower when the HRV was operating continuously for the house with electric baseboard heating, house M.

The effectiveness of the radon reduction was calculated from the indoor radon concentration with the HRV operating compared to the HRV turned off as described in Equation 1:


(1) $$\mathrm{effectiveness}\;\left(\%\right)=100\;\left(1-C_{HRV\_\mathrm{on}}/C_{HRV\_\mathrm{off}}\right)$$

where:

*C_HRV_on_* – average indoor radon concentration with HRV in operation.

*C_HRV_off_* – average indoor radon concentration with HRV off.

The distribution of the effectiveness of continuous HRV operation at reducing radon in the 12 occupied study houses with forced-air furnace heating was reported in Table 3 and shown in the histogram in Supplementary Figure 5. The median (25th–75th percentile) effectiveness was 39% (29–50) overall, with similar radon reductions for the lower and upper floors, at 43% (26–49) and 46% (22–52), respectively. The highest average radon reduction of 80% was observed in the occupied bungalow with electric baseboard heating and an independently ducted HRV installed, having a radon reduction of 74% in the basement and 91% on the upper (main) floor. An average effectiveness of 54% for radon reduction on the lower floor of the energy efficient NRC twin houses was observed for the ventilation system operated continuously at high fan speed; a reduction of 65% was observed in the slightly more airtight twin house, CCHT West, and a reduction of 43% for CCHT East (Supplementary Figure 6).

**Table 3:** HRV effectiveness in occupied study houses with forced-air furnace heating.

Radon reduction	House average (%)	Lower floor (%)	Upper floor (%)
Median	39	43	46
25th percentile	29	26	22
75th percentile	50	49	52
Minimum	20	20	14
Maximum	56	57	58

**Figure 3:**
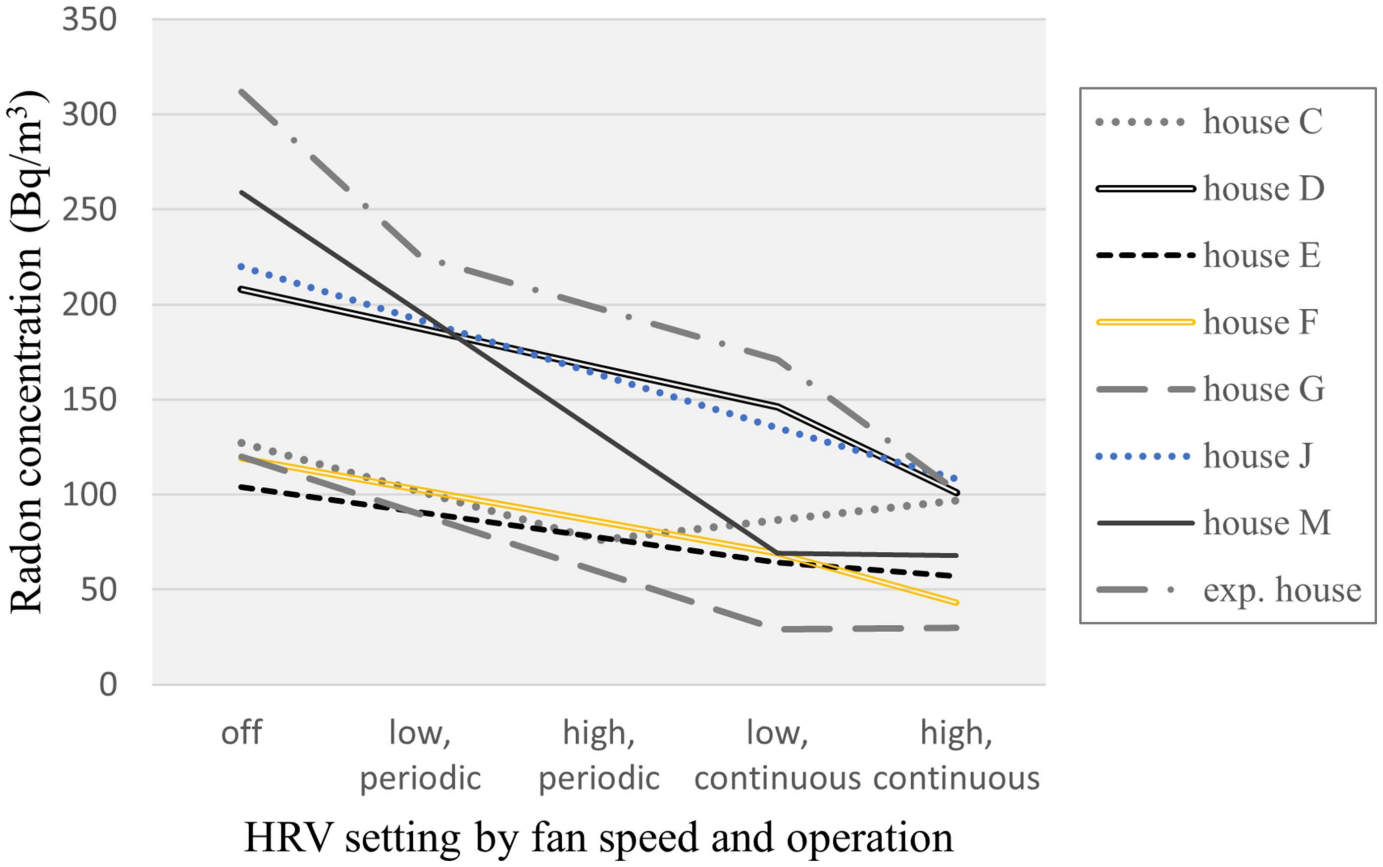
Trends in radon concentration by HRV setting - fan speed and operation.

The trends in lower floor radon concentration by HRV setting were plotted in Figure 3, for the seven occupied study houses and the small NRC experimental house in which more than two HRV settings were assessed. An overall trend of higher effectiveness in radon reduction was observed for continuous than periodic fan operation, and for higher than lower fan speed, although in some houses there was little difference in indoor radon concentration between lower or higher fan speed for continuous HRV operation. While the radon concentrations measured on the upper floor in these houses were all somewhat reduced compared to the lower floor, similar trends were evident for the radon reduction by HRV setting (Supplementary Table 1).

**Figure 4:**
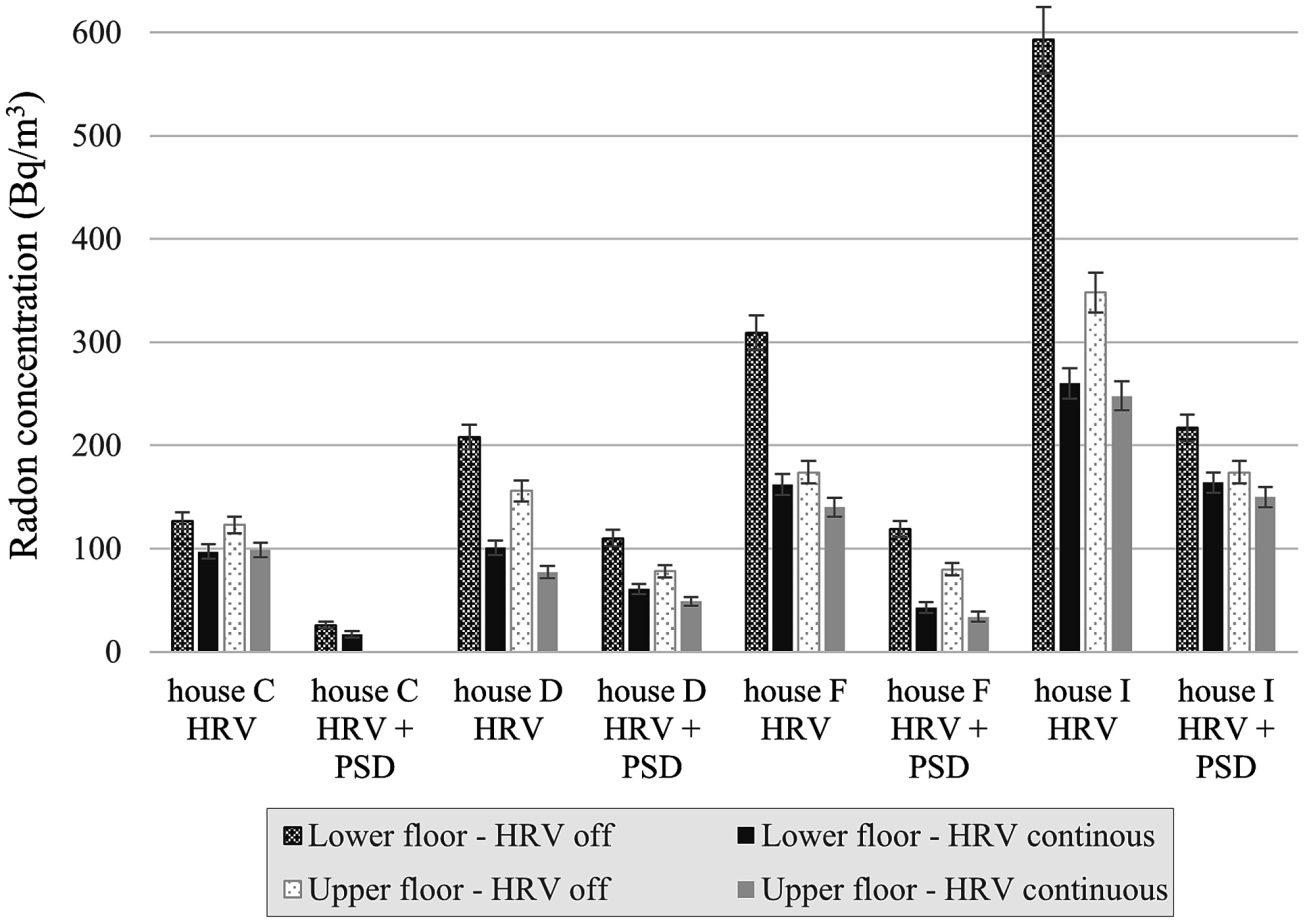
Radon concentrations in study houses with HRV and HRV + PSD, by floor and HRV status.

The indoor radon concentrations measured when the HRV was operated independently and in combination with the PSD system for four field study houses were presented in Figure 4 and Supplementary Table 2. The HRV system reduced the indoor radon concentration in each study house for both scenarios, and the indoor radon concentrations were much lower when the PSD system was operating. No overall trend was evident; while a somewhat higher effectiveness in radon reduction was observed on each floor with the operation of both the HRV and PSD systems in three houses, the opposite was observed in the fourth house. In study house I, both a PSD and an HRV operated continuously were required to reduce the radon concentration below the Health Canada guideline value of 200 Bq/m^3^. The median radon reduction on the lower floor was 40% for continuous HRV operated both independently and in combination with a PSD system.

### 3.2 Mechanical exhaust ventilation, depressurization and indoor radon in CCHT twin houses

**Figure 5:**
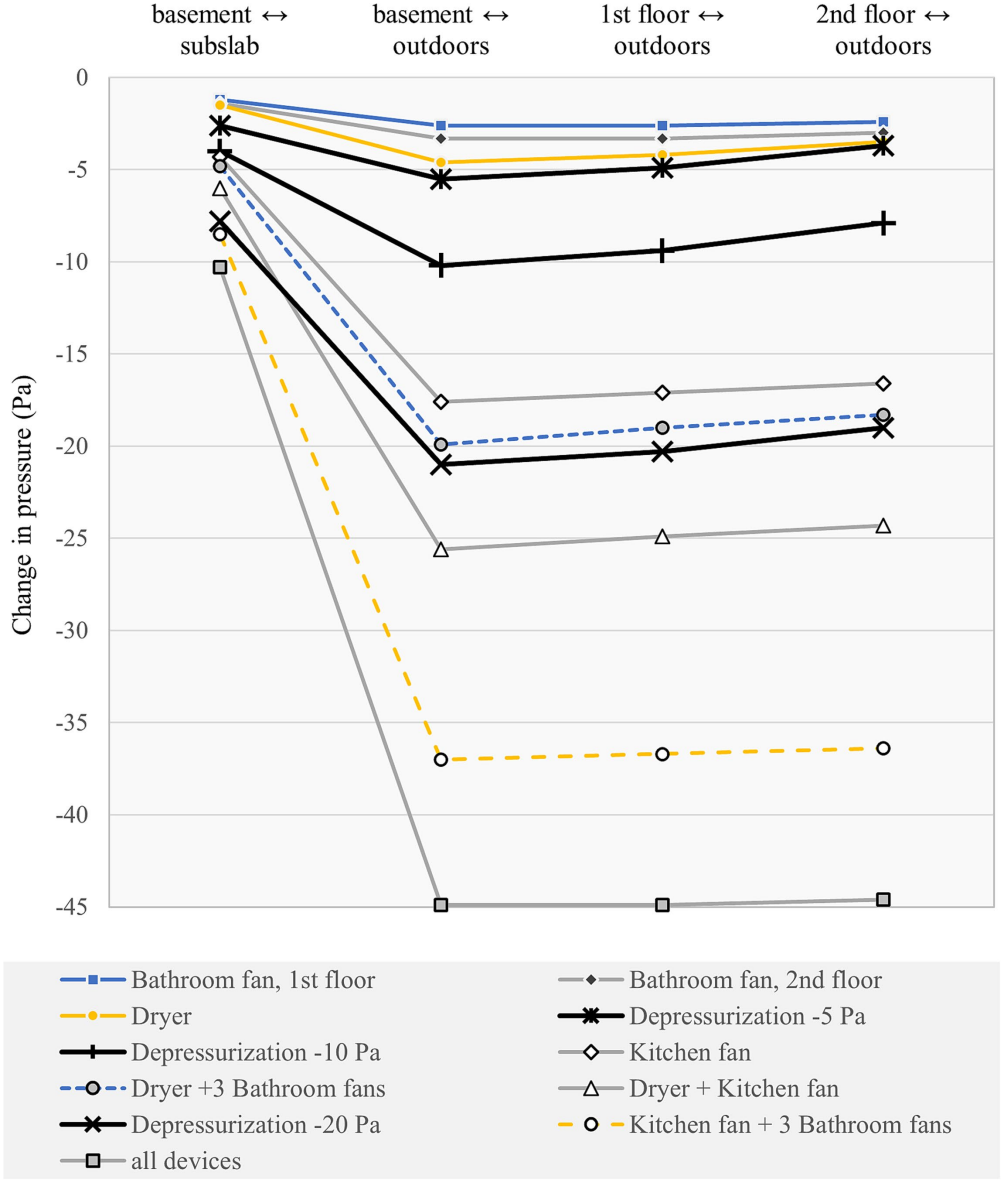
Change in pressure by location in CCHT duplex for mechanical exhaust devices and duct blaster operation.

The continuous operation of mechanical exhaust ventilation devices in NRC’s full size energy efficient twin house increased the depressurization of the basement relative to the subslab by −1 to −10 Pa, and of the indoors relative to the outdoors by −3 to −45 Pa. The range of depressurization resulting from the use of individual mechanical exhaust devices (Table 4) in CCHT West was spanned by the use of a duct blaster to induce three levels of depressurization (−5 Pa, −10 Pa, and −20 Pa) in CCHT East (Table 5), as shown in Figure 5. The individual operation of either the bathroom fan on the main floor, or the bathroom fan on the second floor, or the clothes dryer increased the depressurization of the main floor relative to outdoors by an average of −3 to −5 Pa; the corresponding increase in depressurization of the basement relative to the subslab ranged from −1 to −3 Pa. The operation of either the range hood, or the range hood in combination with the clothes dryer, or the clothes dryer in combination with all three bathroom fans increased the depressurization of the main floor relative to outdoors by −17 to −25 Pa; the corresponding increase in depressurization of the basement relative to the subslab ranged from −4 to −7 Pa. The operation of the range hood with the clothes dryer and all three bathroom fans increased the depressurization of the main floor relative to outdoors by −45 Pa, and of the basement relative to the subslab by −10 Pa. Similar increases in depressurization in CCHT West were observed for both locations of clothes dryer, whether in the basement or on the second floor (Supplementary Table 3).

**Table 4:** Depressurization of CCHT West during operation of mechanical exhaust devices.

Exhaust devices operating in CCHT West	CCHT W Δp basement relative to subslab (Pa)	CCHT W Δp basement relative to outdoors (Pa)	CCHT W Δp1st floor relative to outdoors (Pa)	CCHT W Δp2nd floor relative to outdoors (Pa)
1st floor bathroom fan	−1.2	−2.6	−2.6	−2.4
2nd floor bathroom fan	−1.4	−3.3	−3.3	−3.0
clothes dryer on 2nd floor	−1.5	−4.6	−4.2	−3.5
Range hood	−4.3	−17.6	−17.1	−16.6
Clothes dryer +3 bathroom fans	−4.8	−19.9	−19.0	−18.3
Range hood + clothes dryer	−6.0	−25.6	−24.9	−24.3
Range hood +3 bathroom fans	−8.5	−37.0	−36.7	−36.4
Range hood + clothes dryer +3 bathroom fans	−10.3	−45.2	−45.0	−44.6

**Table 5:** Depressurization of CCHT East using the duct blaster.

Duct blaster Δp CCHT East (Pa)	Recirculation fan in CCHT East	CCHT East Δp basement relative to subslab (Pa)	CCHT East Δp basement relative to outdoors (Pa)	CCHT East Δp1st floorrelative to outdoors (Pa)	CCHT East Δp2nd floorrelative to outdoors (Pa)
−5	Off	−2.6	−5.5	−4.9	−3.7
−10	Off	−4.0	−10.2	−9.4	−7.9
−20	Off	−7.8	−21.0	−20.3	−19.0
−5	On	−2.5	−4.8	−4.4	−3.4
−10	On	−4.6	−11.2	−10.6	−9.3
−20	On	−7.3	−18.9	−18.4	−17.2

Small decreases in pressure measured in CCHT East, ranging from about −1 to −3 Pa, showed an effect from the operation of the mechanical exhaust devices in the twin house, CCHT West (Supplementary Table 4). Similar decreases in pressure of −1 to −2 Pa were observed in CCHT West when the main floor of the twin house, CCHT East, was depressurized relative to the outdoors using the duct blaster (Supplementary Table 5). The relative change in depressurization in the twin house was stronger between the basement and the subslab than between the indoors and the outdoors. For the individual operation of the 1st floor bathroom, the 2nd floor bathroom or the clothes dryer in CCHT West, the increase in depressurization of the basement relative to the subslab ranged from −1.2 to −1.5 Pa in CCHT West and from −0.9 to −1.0 in CCHT East. The induced depressurization of −5 Pa of the main floor relative to the outdoors using the duct blaster in CCHT East resulted in a depressurization of the basement relative to the subslab of −2.5 Pa in CCHT East and −0.7 Pa in CCHT West.

**Figure 6:**
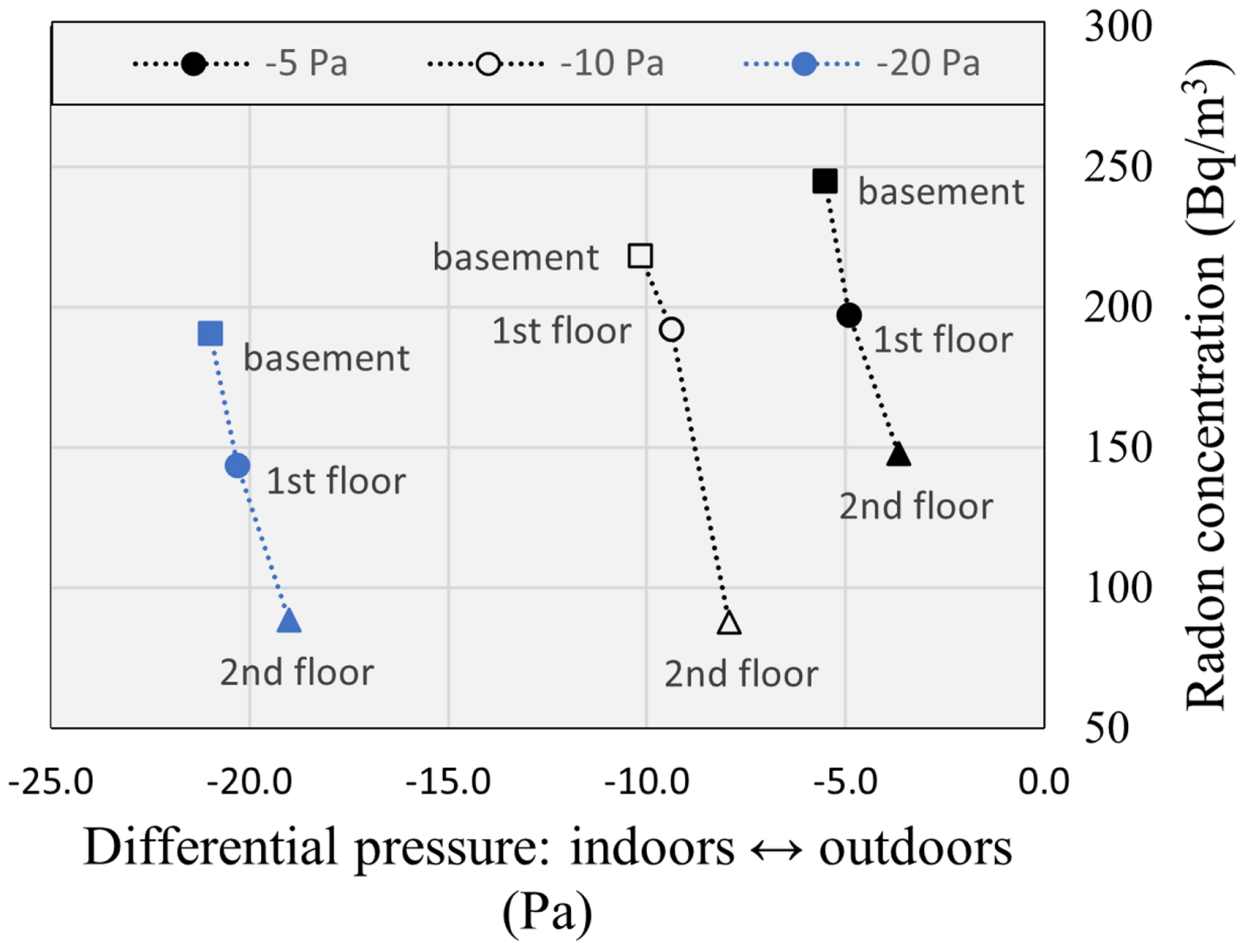
Radon concentration and differential pressure measured in CCHT East by floor during depressurization testing using a duct blaster located on the first (ground) floor with furnace and recirculation fan off.

**Figure 7:**
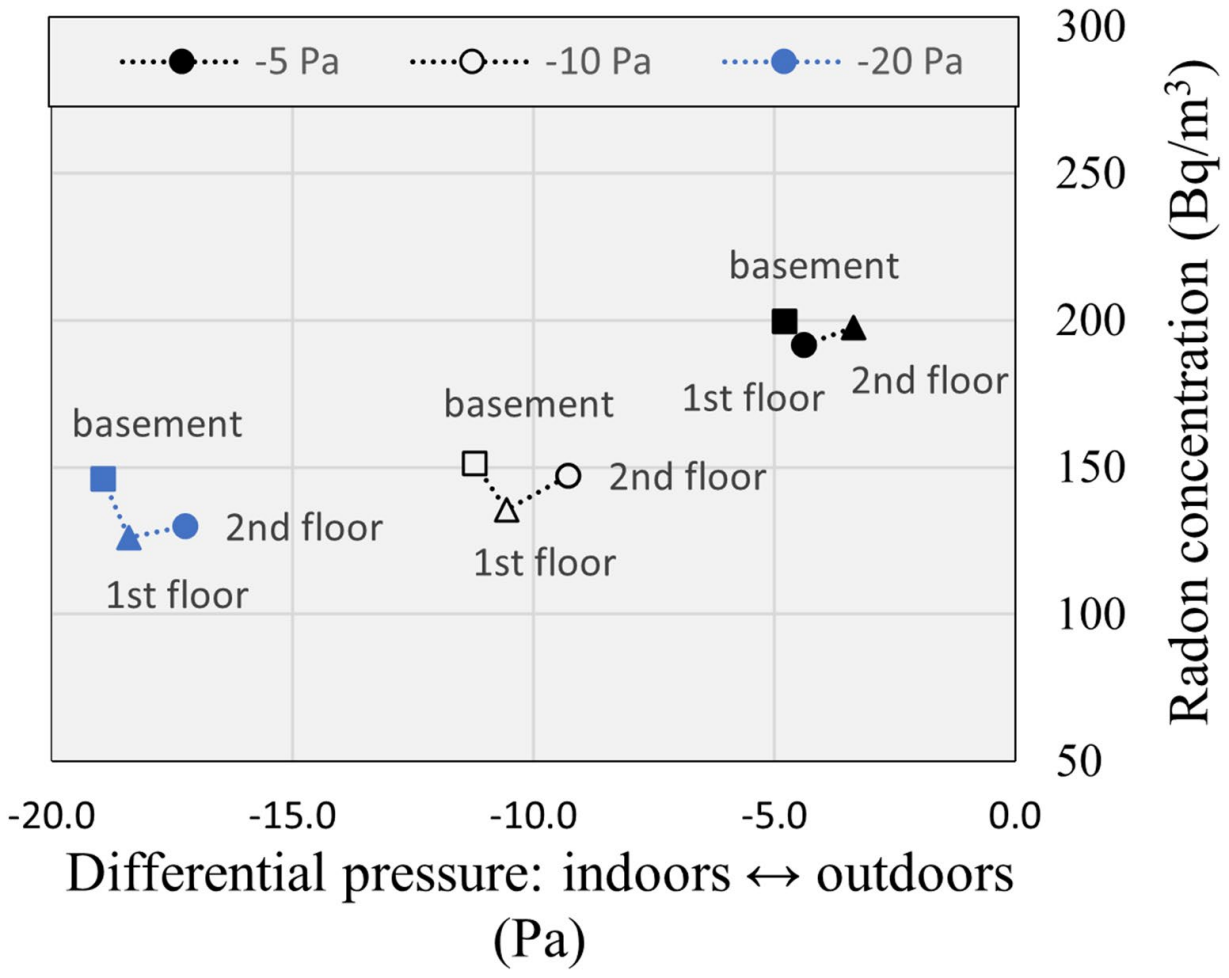
Radon concentration and differential pressure measured in CCHT East by floor during depressurization testing using a duct blaster located on the first (ground) floor with furnace and recirculation fan on.

The depressurization of the basement relative to the subslab is the driving force that leads to the infiltration of soil radon gas across the foundations, although it is the depressurization of the indoors relative to the outdoors that is typically measured in the community. The indoor radon concentration measured on each floor in CCHT East, corresponding to the increased depressurization of the main floor relative to outdoors by −5 Pa, −10 Pa, and −20 Pa using the duct blaster, was plotted in Figures 6, 7, and presented in Supplementary Table 6. The basement indoor radon concentration was plotted versus the increase in depressurization of the basement relative to the subslab in Supplementary Figure 7. The radon concentration on each floor was highest for the lowest depressurization scenario of −5 Pa relative to outdoors, and the highest radon concentration for each depressurization scenario was in the basement with the recirculation fan off. Stratification of indoor radon was evident when the furnace and the recirculation fan were turned off, as shown in Figure 6, being highest in the basement and lowest on the second floor for each depressurization scenario. It is expected that building depressurization would have resulted in soil radon gas infiltration across the foundations, increasing the radon concentration in the basement, and infiltration of outdoor air on the upper floor, diluting the radon concentration upstairs. The radon concentration level varied less between floors when the recirculation fan was turned on, as shown in Figure 7. The indoor radon concentrations measured in CCHT West for each depressurization scenario evaluated in CCHT East using the duct blaster are presented in Supplementary Table 7 (the recirculation fan was turned on in CCHT West). The radon concentrations were all lower in CCHT West than in CCHT East. The highest indoor radon concentrations in CCHT West were also measured at the lowest induced depressurization of -5 Pa in CCHT East using the duct blaster, and the basement radon concentration was highest in CCHT West when the recirculation fan in CCHT East was operating.

The average radon concentration in the energy efficient CCHT twin duplexes was compared in Table 6 for each depressurization scenario induced in CCHT East using the duct blaster, with and without the recirculation fan operating. The highest average indoor radon concentrations in CCHT East occurred for an increased depressurization relative to the outdoors of −5 Pa, at 194 Bq/m^3^ and 197 Bq/m^3^ (recirculation fan off and on). The average indoor radon concentrations in CCHT East decreased as the degree of depressurization increased, and was lowest at 138 and 134 Bq/m^3^ (recirculation fan off and on) for a depressurization of −20 Pa relative to outdoors, and were consistently higher than in the adjacent duplex CCHT West. The relative increase in average indoor radon in CCHT East relative to CCHT West resulting from the use of the duct blaster ranged from 1.6 to 2.5 when the recirculation fan was off. A larger impact on the adjacent twin house was evident when the recirculation fan in CCHT East was operating, for which higher indoor radon concentrations in CCHT West were measured.

**Table 6:** Average radon concentration in CCHT West and CCHT East during depressurization of CCHT East.

Recirculation Fan CCHT West	Radon CCHT West average (Bq/m^3^)	Recirculation Fan CCHT East	Depressurization of CCHT East (Pa)	Radon CCHT East average (Bq/m^3^)	Ratio Radon CCHT East to West
On	123	Off	−5	194	1.6
On	92	Off	−10	163	1.8
On	55	Off	−20	138	2.5
On	161	On	−5	197	1.2
On	76	On	−10	145	1.9
On	76	On	−20	134	1.8

**Figure 8:**
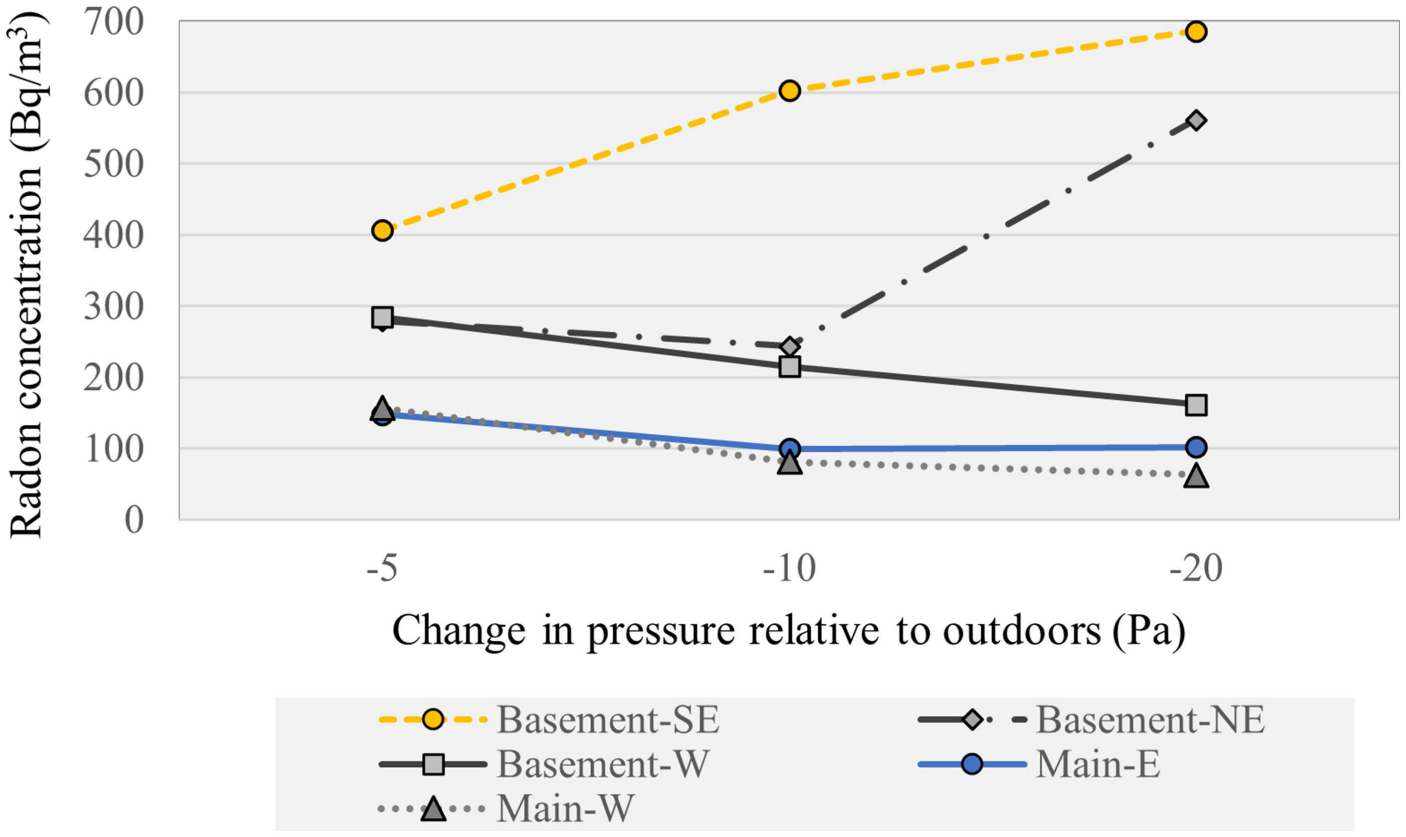
Radon concentration measured in the NRC experimental house under depressurization relative to outdoors.

For the testing conducted in the older NRC experimental house, depressurization of the basement relative to the subslab increased with depressurization of the indoors relative to the outdoors, of −5 Pa, −10 Pa and −20 Pa induced by the exhaust depressurization fan (Supplementary Table 8). The indoor radon concentrations are plotted in Figure 8 and presented in Supplementary Table 9. The indoor radon increased in the enclosed basement rooms (South East and North East) with increasing depressurization of the basement relative to the subslab, suggesting increased soil radon infiltration across the foundations. By contrast, decreasing radon concentrations were observed with increasing indoor depressurization in the upstairs rooms and in the basement-West room connected to the upstairs by an open riser staircase, suggesting increased infiltration of outdoor air across the above ground building envelope. The change in indoor radon varied with the measurement location within the building in the older NRC experimental house, remaining similar by level of depressurization for the building overall.

## 4 Discussion

Balanced mechanical ventilation with heat/energy recovery reduced the moderate initial indoor radon concentrations in the occupied field study houses with forced-air furnace heating by a median effectiveness of 39% (25th–75th percentile: 25th–75th percentile) overall when operated continuously at the high fan speed. The median radon reductions were similar for the lower and upper floors, at 43% (26–49) and 46% (22–52), respectively. An overall trend of higher reduction effectiveness for continuous than periodic fan operation, and for higher than lower fan speed, was observed in the study houses in which more than two HRV settings were assessed. In addition to the fan speed and operation of the HRV, the main factors affecting the effectiveness of radon reduction with HRV and contributing to the variation between different study houses would include the airtightness of the house, with mechanical ventilation having a greater impact on more airtight houses, and the volume of the conditioned space in the house, which determines the proportion of the indoor air exchanged by the mechanical ventilation system. A median radon reduction on the lower floor of 40% was observed for both scenarios in study houses that had HRV and PSD systems. The operation of the HRV system always reduced the indoor radon concentration, whether used by itself or in combination with a PSD system.

The continuous operation of mechanical exhaust ventilation devices in an NRC full-size energy efficient duplex increased the depressurization of the basement relative to the subslab by −1 to −10 Pa, and of the indoors relative to the outdoors by −3 to −45 Pa. Radon concentrations increased with sustained depressurization in both the NRC full-size energy efficient twin duplexes and the older, less airtight experimental house. Indoor radon concentrations measured at increasing depressurization in the energy efficient twin house, using a duct blaster to induce depressurization of the indoors relative to the outdoors by −5 Pa, −10 Pa, and −20 Pa, showed that the highest radon concentration occurred on each floor for the lowest depressurization scenario of −5 Pa. The stratification of indoor radon was evident when the recirculation fan was turned off, with the highest radon concentration measured in the basement for each depressurization scenario, which would be consistent with soil radon gas infiltration across the foundations, and the lowest on the 2nd floor, consistent with dilution due to infiltration of outdoor air upstairs. The change in indoor radon varied with the measurement location within the building in the older NRC experimental house, increasing in the enclosed basement rooms and decreasing in the rooms upstairs and the basement room connected to the upstairs by an open staircase. Basements tend to be occupied spaces in Canada that include bedrooms and family rooms.

This research suggests that radon infiltration even at depressurization of about −5 Pa may be an issue of concern in all low-rise housing, despite being considered low enough to prevent spillage of combustion products from combustion appliances that do not have direct supply and exhaust vents. In duplexes and townhouses, depressurization from the use of mechanical exhaust devices in one unit may affect adjacent units. The indoor radon concentrations were highest for the increase in depressurization of −5 Pa, with the indoor radon concentrations in the depressurized energy efficient NRC duplex consistently higher than in the adjacent duplex.

The median radon reduction of 39% (25–75th percentiles: 29–50%) measured in this field study of occupied houses was consistent with the results of a Finnish and a Norwegian study. It was reported that indoor radon concentrations were 20–47% lower in houses with mechanical supply and exhaust ventilation systems with heat recovery than in houses with mechanical exhaust only, based on the results of the 2006 national radon survey and the national radon database (6). Similarly, the geometric mean indoor radon concentration was 23–40% lower in houses with mechanical supply and exhaust ventilation systems than in houses with mechanical exhaust only, estimated from new houses in the 2008 national survey and those in the 2016 national survey, respectively (8). A pattern of increased indoor radon concentration with depressurization was reported for blower door testing conducted in an older house in France (17).

Improving the energy efficiency of the housing stock is an important objective for many countries to address climate change and to reduce greenhouse gas emissions. In Canada, emissions from heating, cooling and using the built environment, including housing, were estimated to comprise about 18% of the national greenhouse gas emissions in 2020 (18). A volunteer survey of building characteristics in over 3,233 dwellings recruited in Brittany, France, that also included two-month indoor radon measurements reported that indoor radon concentration was 21% higher in those that had undergone a thermal retrofit (19). Replacement of windows, increased thermal insulation and improved ventilation, individually or in combination, were characterized as thermal retrofits, and it was noted that 72% of the retrofits did not include any ventilation improvements. Including balanced mechanical ventilation with heat/energy recovery with energy retrofits may be an effective solution to prevent indoor radon concentrations from increasing when the airtightness in existing housing is improved.

The observation of similar effectiveness of radon reduction for HRV operation with and without a PSD system operating is consistent with the independent operation of the two systems, where the sub-slab depressurization system reduces the ingress of radon indoors from the soil and the HRV system subsequently reduces indoor radon concentration by dilution. In House I, both a passive radon depressurization system and an HRV were required to reduce the indoor radon concentration below the recommended mitigation threshold of 200 Bq/m^3^ for existing housing in Canada. This result was consistent with Norwegian studies that reported significantly lower indoor radon and separate effects identified in multivariate regression for balanced mechanical supply and exhaust ventilation with heat recovery and for a passive radon depressurization system in houses built after the 2010 building code change in Norway (8, 9).

This field study evaluated the effectiveness of balanced mechanical ventilation with heat recovery at reducing indoor radon in occupied houses in the community, which will help to inform larger-scale implementation in the existing housing stock. The results of this study are applicable to the majority of private housing stock in Canada, with single detached, duplex and townhouse dwellings reported to represent 70% of all private dwellings in the 2021 census (5). The results of this study may also be applicable to apartments in high-rise residential buildings that use a decentralized HRV/ERV system having suite-based fans and ducts that supply and exhaust air from individual units. It has been reported, however, that pressurized corridor ventilation systems are more prevalent in high-rise residential buildings and have tended to under-deliver outdoor air to floors and suites (20). A strength of this study was that month-long indoor radon measurement on both the lower and upper floor was conducted for each HRV scenario sequentially within a season for each study house to minimize the effect of seasonal radon variations. The average effectiveness of radon reduction may be a little higher for houses in this study because it was determined from indoor radon concentrations measured after the recent installation and balancing by a certified ventilation contractor in each study house with the HRV system operated continuously at the high fan speed. Regular maintenance in addition to balancing after installation is required for HRVs to continue to operate effectively, with significant problems ranging from poor ventilation to complete system failure reported for balanced mechanical ventilation systems installed in the far North in Canada (21). It was noted that the fewest problems observed for HRVs was in the city of Whitehorse, which required third-party commissioning/verification following installation.

The current study was limited to houses with only moderate initial radon concentrations, those below about 300 Bq/m^3^, and houses such as study house I with higher initial radon may also require active soil depressurizations systems to reduce indoor radon exposures below a mitigation threshold recommended for existing housing. It was not possible to measure the ventilation rates using tracer gas testing in the occupied study houses due to limitations regarding in-person visits during the COVID-19 pandemic. In addition, the majority of the houses recruited into this field study had forced-air furnace heating, and effectiveness of radon reduction may differ for HRVs installed in housing with alternative heating systems. While the scope of this study was restricted to the impact of HRV/ERV on indoor radon, it would be useful to evaluate its impact on other indoor air parameters such as carbon dioxide concentration, humidity, and fine particulate exposures in future studies.

## 5 Conclusion

Balanced mechanical ventilation with heat/energy recovery was shown to be an effective radon control strategy in a field study of occupied houses in the community, characterized by a moderate initial indoor radon concentration. The median radon reduction effectiveness was 39% for HRVs operated continuously with the fan at the high speed setting in the 12 houses where it was connected directly to the forced-air furnace heating systems. Higher reductions in radon were observed for the operation of the HRV/ERVs in the field study house with electric baseboard heating and in the more airtight energy efficient NRC full size twin houses. The sustained operation of mechanical exhaust ventilation devices, however, increased the depressurization and the indoor radon concentration in the full-size energy efficient NRC twin duplexes. The highest indoor radon concentration was measured in the basement for each depressurization scenario, and there was some evidence of depressurization in one duplex affecting the adjacent duplex. The average indoor radon concentration in the energy efficient twin duplex under depressurization was roughly double that in the adjacent duplex for each depressurization scenario. The highest indoor radon concentrations were observed at the lowest depressurization of −5 Pa, which corresponded to the operation of a single mechanical exhaust device, such as a bathroom fan or a clothes dryer. Improving the energy efficiency of the existing housing stock is a priority in many countries, and the inclusion of balanced mechanical ventilation systems with energy retrofits may be an effective radon control option for existing houses.

## Data Availability

The original contributions presented in the study are included in the article/Supplementary material, further inquiries can be directed to the corresponding author.
