# Supplementary material for: Ventilation approaches and radon control in Canadian houses
Source: Front Public Health. 2025 Apr 10;13:1569494. doi: 10.3389/fpubh.2025.1569494 (PMC12057567; doi:10.3389/fpubh.2025.1569494)
Supplement: Supplementary file 1 [file Data_Sheet_1.docx]

Supplementary Material

**Supplementary Tables**

Supplementary Table 1: Trends in radon concentration by HRV setting, on lower and upper floors

| House ID | PSD operating | Radon  HRV  Off  (Bq/m^3^) | **radon**  HRV  20/40  low speed  (Bq/m^3^) | **radon**  HRV  20/40  high speed  (Bq/m^3^) | radon  HRV  continuous  low speed  (Bq/m^3^) | radon  HRV  continuous  high speed  (Bq/m^3^) |
| --- | --- | --- | --- | --- | --- | --- |
| C | 🗶 | 127 | - | 76 |  | 97 |
| D | 🗶 | 208 | - | - | 146 | 101 |
| E | 🗶 | 104 | - | - | 64 | 57 |
| F | ✓ | 119 | - | - | 69 | 43 |
| G | 🗶 | 120 | - | - | 29 | 30 |
| J | 🗶 | 220 | - | - | 135 | 108 |
| M | 🗶 | 259 | - | - | 69 | 68 |
| P | 🗶 | 312 | 225 | - | 171 | 102 |

Supplementary Table 2: initial radon concentrations and radon reduction effectiveness on upper and lower floors for continuous HRV, with & without passive radon mitigation system operating

| House ID | Time period | PSD operating | **Initial radon**  **L floor**  HRV off  (Bq/m^3^) | **Reduction effectiveness**  **L floor**  HRV continuous | **Initial radon**  **U floor**  HRV off  (Bq/m^3^) | **Reduction effectiveness**  **U floor**  HRV continuous |
| --- | --- | --- | --- | --- | --- | --- |
| C | 2021-01-16/  2021-03-15 | 🗶 | 127 ± 8 | 24% | 123 ± 8 | 20% |
| C | 2021-04-19/  2021-06-15 | ✓ | 26 ± 3 | 35% | - | - |
| D | 2021-03-07/  2021-05-07 | 🗶 | 146 ± 9 | 31% | 128 ± 8 | 40% |
| D | 2021-11-06/  2022-01-01 | ✓ | 110 ± 8 | 45% | 78 ± 6 | 37% |
| F | 2021-02-17/  2021-04-19 | 🗶 | 309 ± 17 | 48% | 174 ± 11 | 20% |
| F | 2021-05-19/  2021-07-26 | ✓ | 119 ± 8 | 64% | 80 ± 6 | 58% |
| I | 2021-09-28/  2022-03-01 | 🗶 | 593 ± 32 | 56% | 348 ± 19 | 29% |
| I | 2021-10-29/  2021-12-24 | ✓ | 217 ± 13 | 24% | 174 ± 11 | 14% |

Note: the indoor radon concentrations measured in House I during both shoulder seasons for different HRV settings (fall and spring) were included in this table due to the relatively small number of homes used to compare the effectiveness of radon reduction using HRV with and without the PSD system operating.

Supplementary Table 3: Depressurization in CCHT West by location of dryer

| Exhaust Devices Operating in CCT West | CCHT West  Δp  basement relative to subslab  (Pa) | CCHT West  Δp  basement relative to outdoors  (Pa) | CCHT West  Δp  1^st^ floor relative to outdoors  (Pa) | CCHT West  Δp  2^nd^ floor relative to outdoors  (Pa) |
| --- | --- | --- | --- | --- |
| Testing: Summer 2024 |  |  |  |  |
| clothes dryer in basement | -1.3 | -6.2 | -6.4 | -6.7 |
| clothes dryer in basement + 3 bathroom fans | -4.9 | -21.9 | -22.5 | -21.7 |
| clothes dryer in basement + 3 bathroom fans + range hood | -10.8 | -48.7 | -49.7 | -49.9 |
| clothes dryer on 2^nd^ floor | -1.3 | -6.0 | -6.3 | -6.4 |
| clothes dryer on 2^nd^ floor + 3 bathroom fans | -5.2 | -21.9 | -22.4 | -22.5 |
| clothes dryer on 2^nd^ floor + 3 bathroom fans + range hood | -11.3 | -49.3 | -50.1 | -50.5 |
| Testing: Fall 2021 |  |  |  |  |
| clothes dryer on 2^nd^ floor | -1.5 | -4.6 | -4.2 | -3.5 |
| clothes dryer on 2^nd^ floor + 3 bathroom fans | -4.8 | -19.9 | -19 | -18.3 |
| clothes dryer on 2^nd^ floor + 3 bathroom fans + range hood | -10.3 | -45.2 | -45 | -44.6 |

Supplementary Table 4: Depressurization of CCHT East during operation of mechanical exhaust devices in CCHT West

| Exhaust Device Operating in M24 F | CCHT East  Δp  basement relative to subslab  (Pa) | CCHT East Δp  basement relative to outdoors  (Pa) | CCHT East  Δp  1^st^ floor relative to outdoors  (Pa) | CCHT East  Δp  2^nd^ floor relative to outdoors  (Pa) |
| --- | --- | --- | --- | --- |
| 1^st^ floor bathroom fan | -0.9 | -0.4 | -0.4 | 0.0 |
| 2^nd^ floor bathroom fan | -0.9 | -0.7 | -0.8 | -0.3 |
| clothes dryer on 2^nd^ floor | -1.0 | -1.5 | -1.1 | 0.1 |
| range hood | -0.6 | -1.5 | -1.4 | -0.6 |
| clothes dryer + 3 bathroom fans | -0.7 | -2.1 | -2.2 | -0.7 |
| range hood + clothes dryer | -0.5 | -2.3 | -2.1 | -1.3 |
| range hood + 3 bathroom fans | -0.4 | -2.8 | -2.5 | -1.4 |
| range hood + clothes dryer + 3 bathroom fans | -0.2 | -3.1 | -3.2 | -2.3 |

Supplementary Table 5: Depressurization of CCHT West using duct blaster in CCHT East

| Duct blaster  Δp  CCHT East  (Pa) | Recirculation fan in  CCHT East | CCHT West  Δp  basement relative to subslab  (Pa) | CCHT West Δp  basement relative to outdoors  (Pa) | CCHT West  Δp  1^st^ floor relative to outdoors  (Pa) | CCHT West  Δp  2^nd^ floor relative to outdoors  (Pa) |
| --- | --- | --- | --- | --- | --- |
| -5 | Off | -0.7 | -1.7 | -0.6 | 0.5 |
| -10 | Off | -0.6 | -2.1 | -0.9 | 0.5 |
| -20 | Off | -0.5 | -2.6 | -1.4 | -0.1 |
| -5 | On | -0.6 | -1.2 | -0.4 | 0.5 |
| -10 | On | -0.6 | -2.0 | -0.9 | 0.1 |
| -20 | On | -0.4 | -2.1 | -1.1 | -0.1 |

Supplementary Table 6: Radon concentrations in CCHT East for duct blaster depressurization of CCHT East

| Depressu-rization of CCHT East  (Pa) | Recircula-tion Fan CCHT East | Radon CCHT East  basement  (Bq/m^3^) | Radon CCHT East  1^st^ floor  (Bq/m^3^) | Radon CCHT East  2^nd^ floor  (Bq/m^3^) | Radon CCHT East  Average  (Bq/m^3^) |
| --- | --- | --- | --- | --- | --- |
| -5 | Off | 245 | 197 | 148 | 194 |
| -10 | Off | 218 | 192 | 88 | 163 |
| -20 | Off | 191 | 143 | 89 | 138 |
| -5 | On | 200 | 192 | 198 | 197 |
| -10 | On | 151 | 136 | 147 | 145 |
| -20 | On | 146 | 126 | 130 | 134 |

Supplementary Table 7: Radon concentrations in CCHT West for duct blaster depressurization of CCHT East

| Depressu-rization  of CCHT East  (Pa) | Recircula-tion Fan CCHT East | Radon CCHT West  basement  (Bq/m^3^) | Radon CCHT West  1^st^ floor  (Bq/m^3^) | Radon CCHT West  2^nd^ floor  (Bq/m^3^) | Radon CCHT West  average  (Bq/m^3^) |
| --- | --- | --- | --- | --- | --- |
| -5 | Off | 136 | 124 | 112 | 123 |
| -10 | Off | 101 | 93 | 84 | 92 |
| -20 | Off | 59 | 56 | 51 | 55 |
| -5 | On | 169 | 166 | 150 | 161 |
| -10 | On | 84 | 80 | 65 | 76 |
| -20 | On | 79 | 78 | 71 | 76 |

Supplementary Table 8: Depressurization testing in NRC experimental test hut

| Duct Blaster Δp: indoors relative to outdoors  (Pa) | Experimental test hut Δp:  basement relative to subslab  (Pa) |
| --- | --- |
| -5 | -0.1 |
| -10 | -0.9 |
| -20 | -1.6 |

Supplementary Table 9: Radon concentrations during depressurization of experimental test hut

| Depressurization  of test hut  (Pa) | Radon basement  SE  (Bq/m^3^) | Radon  Basement  NE  (Bq/m^3^) | Radon  Basement  W  (Bq/m^3^) | Radon  main floor  E  (Bq/m^3^) | Radon  main floor  W  (Bq/m^3^) |
| --- | --- | --- | --- | --- | --- |
| -5 | 406 | 279 | 284 | 148 | 157 |
| -10 | 602 | 244 | 215 | 99 | 81 |
| -20 | 687 | 562 | 162 | 102 | 63 |

**Supplementary Figures**


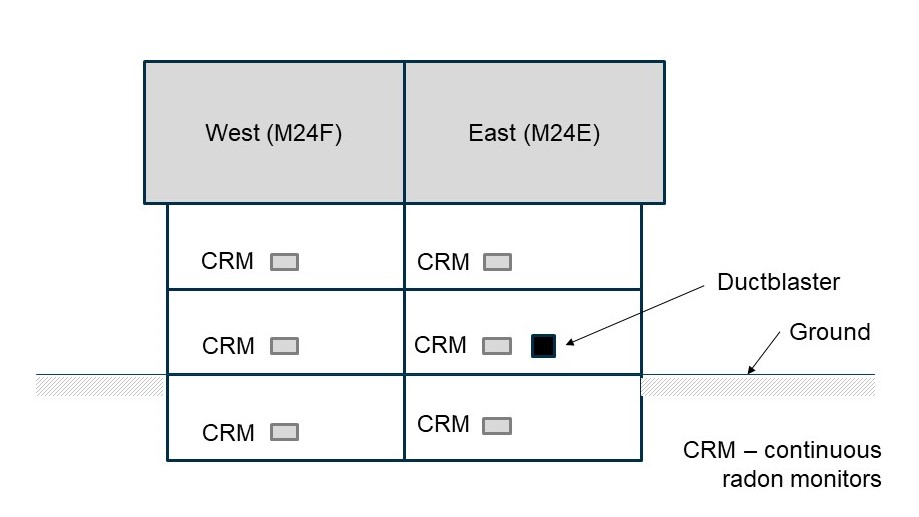


Supplementary Figure 1: CCHT twin duplexes, locations of continuous radon monitors and duct blaster


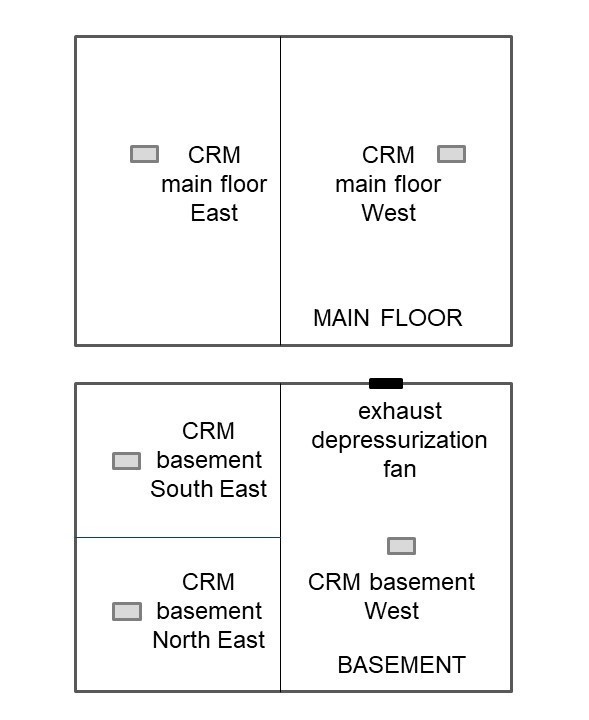


Supplementary Figure 2: NRC experimental hut, location of continuous radon monitors and exhaust depressurization fan


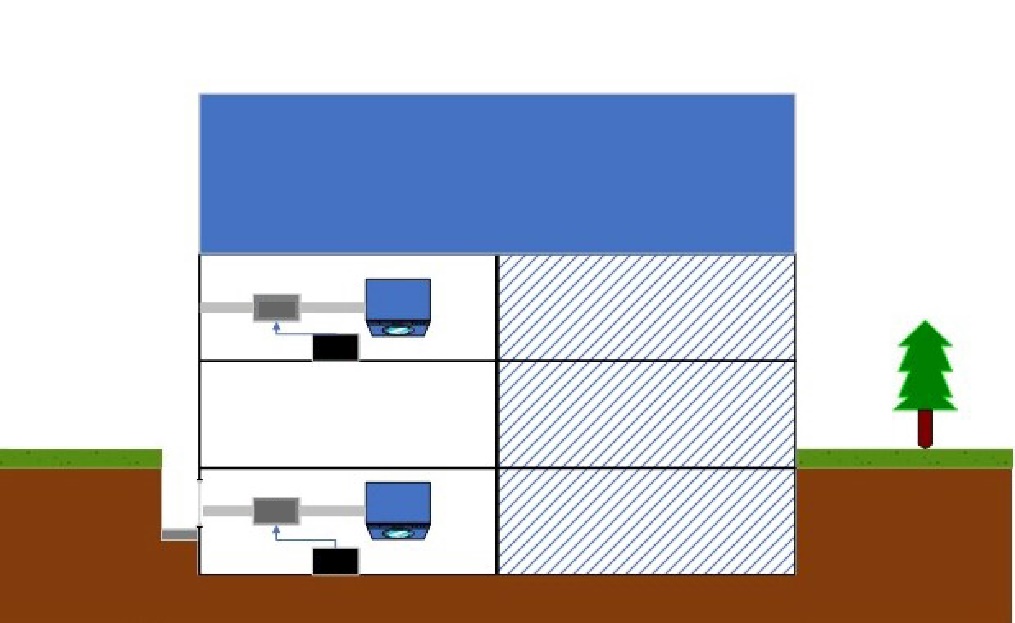


Supplementary Figure 3: CCHT, locations of clothes dryer evaluated


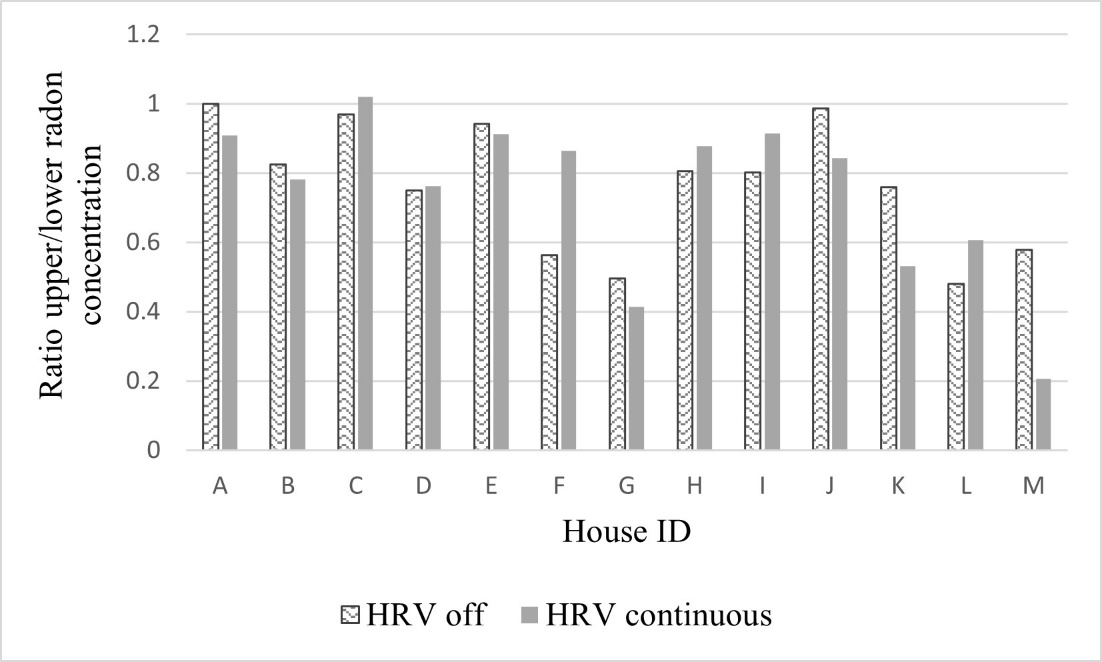


Supplementary Figure 4: Ratio of upper/lower floor radon concentration in occupied study houses


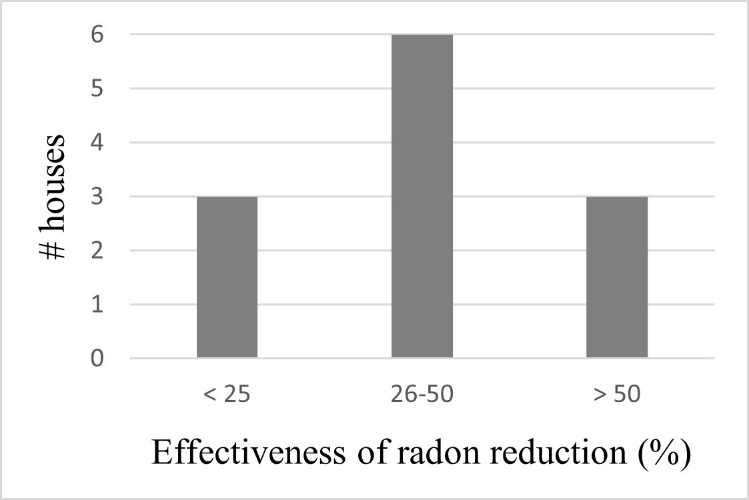


Supplementary Figure 5: histogram of effectiveness of radon reduction in field study houses with forced-air furnace heating


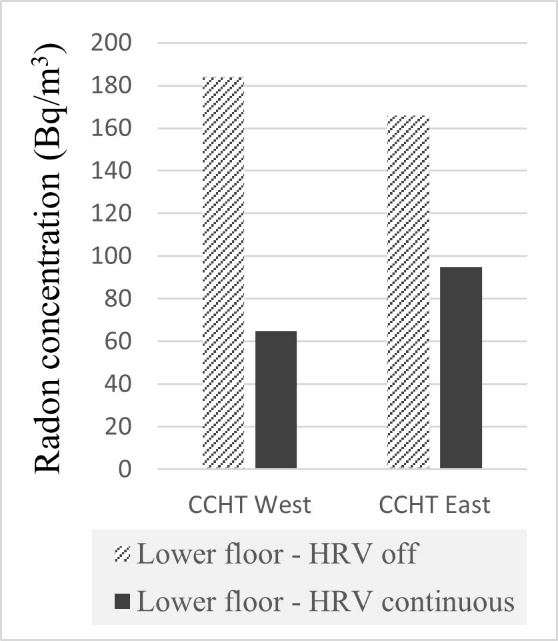


Supplementary Figure 6: radon concentrations in energy efficient NRC twin duplex houses by HRV status


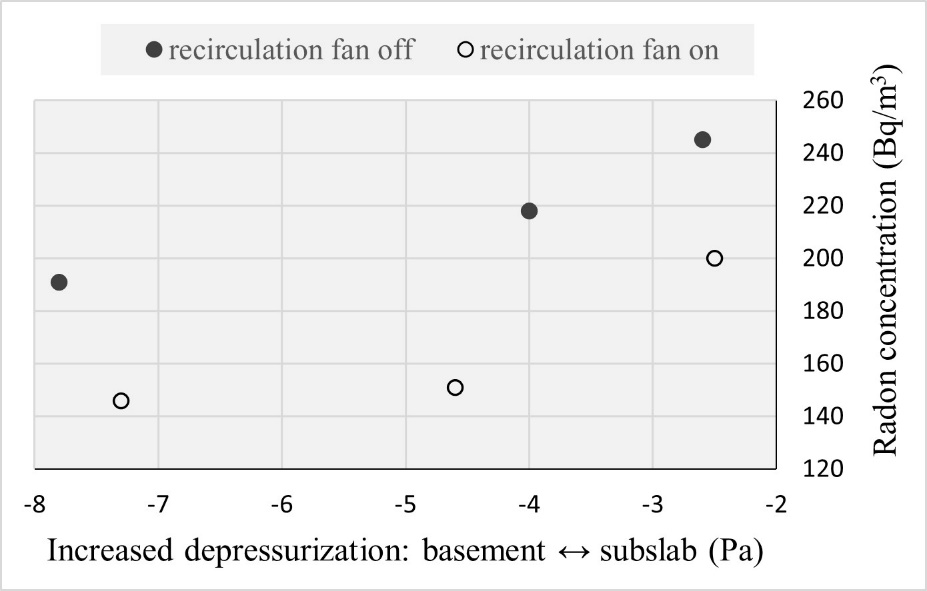


Supplementary Figure 7: Basement radon concentration and change in pressure of the basement relative to the subslab, with recirculation fan on and off
